# How perceived discrimination and trust dynamics influence social integration in acculturation and reacculturation: the case of Chinese international student returnees

**DOI:** 10.3389/fpsyg.2025.1597967

**Published:** 2025-05-15

**Authors:** Tam-Tri Le, Ruining Jin

**Affiliations:** ^1^Independent Researcher, Ho Chi Minh City, Vietnam; ^2^Institute of Higher Education, Beijing University of Technology, Beijing, China; ^3^Civil, Commercial and Economic Law School, China University of Political Science and Law, Beijing, China

**Keywords:** generalized trust, personalized trust, discrimination, acculturation, returnees

## Abstract

**Background:**

When coming to a new social environment, many people unfortunately are under the mental burden of perceived discrimination. The Chinese international student returnees in the post-COVID-19 era present a special case where they experienced considerable discrimination issues during both the processes of acculturation in the host countries as well as reacculturation after returning to China. This study aims to investigate group-based influences on the impacts of discrimination-related perceptions toward social integration (and reintegration) degrees through different psychological pathways of trust.

**Analysis:**

Employing Bayesian analyses aided by Markov Chain Monte Carlo (MCMC) algorithms on survey data of 1,014 Chinese international student returnees, the study examines the multi-layered influences of perceived discrimination and trust (both generalized and personalized) on social integration.

**Findings:**

We found that in the foreign social environment, interestingly, the degree of acculturation and perceived discrimination has a positive association. Generalized trust positively moderates this association while personalized trust has a negative moderating effect. In the domestic social environment, perceived discrimination is positively associated with the degree of negative mental health induced by concerns about whether to stay in China or emigrate. Generalized trust positively moderates this association, while personalized trust negatively moderates it.

**Conclusion:**

The findings suggest the possible information exchange pathways where different types of trust and group-based perceptions interact.

## 1 Introduction

### 1.1 Acculturation, reacculturation, and acculturative stress

Acculturation refers to both cultural and psychological changes resulting from interactions between different cultural groups and individuals, which encompasses changes in social structures, institutions, cultural practices, and various individual behaviors (Berry, 1997). Reacculturation, on the other hand, refers to the reassimilation stages one goes through to reintegrate into their home culture after residing in a distinct cultural setting for a substantial duration (Gaw, 2000). Reacculturation difficulties often stem from the combined challenges of readjusting to a changed home environment, undergoing personal identity shifts, and experiencing a loss of status, which collectively contribute to psychological disorientation and mental distress for returnees (Adler, 1975; Gaw, 2000; Szkudlarek, 2010).

During acculturation and reacculturation, the experience of difficulties and hardships such as social support loss, self-esteem loss, and identity conflicts can be defined as acculturative stress, which might differ based on the type of acculturating groups, individual characteristics (such as gender, age, and education, social networks, level of social support, and social status) (Berry et al., 1987), and personal experiences such as perceived discrimination (Adler, 1981; Adler, 1975; Revollo et al., 2011; Smart and Smart, 1995).

### 1.2 The noteworthy case of Chinese international students in recent times

The outbreak and the long-lasting impacts of post-COVID-19 have significantly reshaped the acculturation and reacculturation experiences of Chinese international students, causing a tremendous amount of acculturative stress during their overseas studies as well as reintegration back into China. During the initial outbreak of COVID-19, Chinese international students encountered a series of mental, physical, and financial hardships, as they suffered from social isolation and lack of social support, experienced online learning challenges, endured safety and security concerns, along with financial strain and the uncertainty towards changing policy directives and plans (Chen et al., 2024; Wu et al., 2022; Zhang and Chan, 2022). In the post-COVID-19 era, when Chinese international student returnees made their reentry, their life was still impacted by the ramifications of COVID-19, as it has altered the social, economic, and cultural landscapes worldwide. Specifically, in China, numerous socioeconomic crises surfaced—ranging from precipitating fertility rates, sluggish economic momentum, hiking unemployment rates among the youth, and a large scale of emigration composed of highly skilled labor (Bloomberg News, 2023; ICEF, 2023; IMF, 2023; Li, 2023; Wong and Yan, 2023; Wu et al., 2022).

In addition to the abovementioned negative impacts of COVID-19, what’s more concerning is the discriminatory experiences returnees endured in both the host and home country. In the initial outbreak of COVID-19, Chinese international students were discriminated against for their mask-wearing practices (Ma and Zhan, 2022), as well as the “ChinaVirus,” “WuhanVirus” rhetoric promoted and fueled by misleading media and politicians (Ma and Miller, 2021; Zheng et al., 2020). A study comparing Chinese students in the United States before and during the COVID-19 pandemic found that students during the pandemic reported higher levels of perceived discrimination and anxiety, with media portrayal of Chinese individuals negatively partly accounting for increased perceived discrimination (Haft and Zhou, 2021); the anti-Asian rhetoric and American’s nationalism during the Trump administration also fanned the flame, making Chinese international students and the Asian community vulnerable to racism and discriminatory acts (Yu, 2022).

However, Chinese international students and student returnees soon received another wave of discrimination from the domestic end. During the COVID-19 pandemic, Chinese internet was filled with surging nationalism and anti-West populist rhetoric (Catalano and Wang, 2021; Cheng et al., 2022). In this context, returnees were forced to take a stand between China and the West (Tao, 2021), and those who failed to side with China were stigmatized, discriminated against, and even doxed on social media. For example, during hotel quarantine, one returnee requested bottled water but was denied, leading her to believe her human rights were violated (Global Times, 2020), and the video clip quickly spread on Chinese social media, with most Chinese netizens supporting the medical staff and criticizing the returnee for not following COVID-19 prevention guidelines, labeling her as a “giant infant” (Yu, 2021); Similarly, others who defied or questioned quarantine order were also reported to be the victims of discriminatory acts and cyberbullying from cybernationalists including harassment, doxing, or even administrative penalties from the workplace and the government (e.g.: censoring, firing, and deportation) (Jung, 2020; SCMP, 2020).

Yet COVID-19 “double-stigmatization” reflects only a fraction of the discriminations that the Chinese international student returnee population suffered from in both foreign and domestic societies. Before COVID-19, numerous reports have indicated that Chinese international students were victims of discrimination from various sources in foreign countries, including local students, university faculty and staff, and residents, in both covert and overt forms due to reasons such as cultural misunderstanding, prejudice, language barriers, and education biases (Lee et al., 2017; Ruble and Zhang, 2013; Wu, 2015; Xie et al., 2019); unfortunately, because studying overseas is considered a tool for upper social classes to maintain status, and for upper middle classes to gain social mobility (Fan and Cheng, 2018; Wang, 2020), they were also viewed unfavorably by the Chinese public when it comes to their lifestyle and social economic status. For example, in 2012, when two Chinese international students were shot to death in their car during their overseas staying, domestic media coverage focused on the brand of the luxury car they were in when the incident occurred, which stirred public outcry not for the violence, but for the luxury lifestyle this population maintained (Chen, 2012); also, because of the ideological conflicts between China and the West, Chinese international students who sided with the West were usually discriminated against before the “giant infant” discrimination during the COVID-19 (Jin and Wang, 2022; Yu, 2021). In 2017, one Chinese international student was slammed, stigmatized, and even doxed by Chinese netizens for giving a commencement speech praising the higher air quality in the United States than that of China (ChinaDaily, 2017; Reuters, 2017).

Furthermore, in the post COVID-19 era, new forms of discrimination emerged in both home and host country towards Chinese international students. For instance, in 2025, one Chinese international students’ Student Visa was revoked for participating a rally protesting against U.S. diplomatic stance in the Middle East regional conflict (Tribune, 2025). In addition, in April 2025, the U.S. government revoked a large number of Chinese international students’ Student Visa, citing vague reasons such as background checks or minor infractions (AP News, 2025). In the home country front, for example, short videos on social media platforms such as *Douyin, RedNote,* and *BiliBili* targeted certain returnee groups (such as the one-year master’s degree holders), using derogatory terms such as “水硕” (literally translated as counterfeit master’s degree) to cast doubt on their credential due to the length of staying and perceived low difficulty for completing such degrees in the host nation (Lyu, 2022). Even state-run media *People.com* issued the opinion-editorial, calling the public to “be aware of high credential, low competence” overseas counterfeit master’s degree and doctorate degree holders (People.com, 2024). Correspondingly, many public and private employers set rules in hiring practices to invalidate one-year overseas master’s degree and exclude these applicants (Minhang, 2022).

### 1.3 Discrimination and social integration

From a sociological perspective, self-categorization refers to the psychological process through which an individual identifies themselves as part of a specific group with defined social values and characteristics (Turner et al., 1987). According to the notion of social conformity, individuals tend to align their attitudes, opinions, or behavior with those of others, either because others are considered more knowledgeable in the given situation, or because individuals want to be liked and gain and reinforce their group membership (Stallen and Sanfey, 2015). Self-concept is partly shaped by the groups one belongs to, thus conformity may help boost self-esteem and sense of belonging, which can facilitate a psychological reinforcing loop toward social conformity desire. Several well-known experiments (Asch, 1956; Milgram, 1963; Sherif, 1936) have demonstrated that in situations of uncertainty or in order to gain group membership, one tends to conform to the group’s rules and norms, even though these might differ from their own personal values and other groups’ values (Tajfel and Turner, 1979). Based on these perspectives, the discriminations that returnees faced reflected the clashes between the group identities formed by Chinese international students and returnees and the prevailing norms within their home and host societies. This clash can lead to tensions, misunderstandings, and distrust, as individuals struggle to reconcile their changed identities with societal expectations and norms.

However, from the Realistic Group Theory (RCT) perspective, discrimination as a form of hostility can stem from the competition for scarce resources, regardless if it is real or perceived (Berkowitz and Sherif, 1967). In this regard, the discrimination returnees received from both domestic and abroad can be understood as perceived competition over scarce resources, such as jobs, social status, or particularly, public health resources during the COVID-19 pandemic. Such a group-based psychological pattern has been observed in the history of mankind as well as in the social behaviors of other species. Evolutionary sociobiological studies dating back to the early age of humanity indicated that human societies were trying to survive through social cooperation as well as caution toward unestablished social connections, suggested to be survival mechanisms evolved from similar patterns found widely among the animal kingdom (Berns et al., 2005; Boyd and Richerson, 1982; Claidière and Whiten, 2012; Konrad and Morath, 2012; Mahajan et al., 2011; Raihani, 2021).

### 1.4 The role of trust in shaping perceptions towards interpersonal connections

In sociopsychological research, trust can be categorized into two types regarding the interpersonal boundary of the trusted group: generalized trust refers to trust towards individuals in society at large, including strangers; while personalized trust is trust towards individuals with whom one has established relationships (Kramer, 2010; Vuong et al., 2023). Prior studies indicated that people with generalized trust tend to display an attitude of openness and inclusion, which predict more positive interactions with diverse individuals and groups, thus reducing the likelihood of experiencing or perpetuating discrimination (Nannestad, 2008; Uslaner, 2002; Welch et al., 2005); on the other hand, personalized trust is well-documented to be associated with discrimination (Brewer, 1999; Delhey and Newton, 2005; Uslaner, 2017; Yamagishi and Yamagishi, 1994). Yamagishi and Yamagishi (1994)’s work in particular, addresses a critical association between personalized trust and discrimination in the United States and Japan, arguing that personalized trust would promote favoritism toward one’s own group and, therefore can heighten the probability of discriminating against those who are not perceived as part of one’s ingroup. Delhey and Newton (2005)’s cross-national study on social trust also indicated that high personalized trust society might be less likely to tolerate outgroup members, indicating possible associations between personalized trust and discrimination. In the case of Chinese international students, reliance on these two forms of trust has implications for how they perceive and respond to discrimination abroad and at home. Strong personalized trust might protect individuals via close social ties yet also amplify sentiments of “us versus them,” whereas higher generalized trust may buffer discriminatory experiences through a broader sense of social goodwill.

The nuanced difference between perceived foreign and domestic discrimination experience can also be viewed through group collective belief-led ingroup and outgroup discrimination. Ingroup discrimination is often due to internal social hierarchies or sub-group identities within the group (Tajfel, 1978), whereas outgroup discrimination is driven by the categorization of individuals as belonging to an external group, often fueled by stereotypes or perceived competition (Brewer, 1999). For example, the outgroup (nation-based) discrimination abroad towards Chinese international students in the West in recent times might come from the general public’s stereotypical collective belief of the negativity towards mask-wearing practice prior to the outbreak of COVID-19 (Ma and Zhan, 2022), or the misinformation about the origin of COVID-19 (Ma and Miller, 2021; Zheng et al., 2020), among other possible reasons. On the other hand, the collective belief from the Chinese public might consider Chinese returnees as “crazy rich” (Xie et al., 2020), or view returnees’ liberalized or individualized value and ideology prioritization as “giant infants” (Jin and Wang, 2022), resulting in ingroup (social hierarchy) discrimination or even outgroup discrimination (nation-ideology based).

### 1.5 Examining group-based perceptions in information interactions during social integration processes

Considering the gap in the understanding of the relationships between discrimination and social integration in modern transnational contexts, especially among Chinese international student returnees, this study serves as an exploratory investigation into such matters. People’s perceptions in both foreign and domestic environments are the targets of examination in a retrospective manner. For this purpose, the present study contains two analytical models corresponding to how perceived discrimination affects social integration in the host country during the time spent abroad and in China after returning, respectively (please see method section for details). In the context of the foreign environment, social integration is represented by the degree of acculturation, following standard conceptualization in sociocultural adaptation research. However, social reintegration into modern Chinese society after returning from abroad is a unique context. Here, corresponding mental processes can be represented by the degree of concerns and perceived burden over the intent or planning whether to stay in China or emigrate, which reflects inner conflicts about the adaptation to the current domestic environment. Furthermore, generalized trust (society-specific) and personalized trust (person-specific) represent the influences of group-based information compatibility toward social perceptions. The personal focus and strength of information exchange channels reflected by the degree of each type of trust can help shed light on specific directional perception reinforcement. Thus, generalized trust (in the respective environments of either host or home society) and personalized trust are examined for potential moderating influences toward the aforementioned relationships affecting self-perceived social integration. In brief, the current study has two main research objectives:

1. Investigating the associations between the level of social integration and perceived discrimination among Chinese international student returnees during their overseas experience and during their staying period in China after returning.
2. Investigating the possible moderating effects of generalized and personalized trust in both relationships above.

## 2 Methodology

### 2.1 Materials and variables

1,014 Chinese international student returnees participated in the survey. The survey was conducted via Chinese international student returnee public WeChat groups (city-based) in Beijing, Shanghai, Suzhou, Shenzhen, and Guangzhou. The data was collected in October 8, 2023- January 30, 2024. To avoid the “honeymoon” phase’s mitigating impact on acculturative stress (Oberg, 1954), the inclusion criteria for this survey include: (1) participants were born and grew up in China and traveled abroad for educational purposes; (2) participants returned to China and stayed for at least 1 year after their studies abroad; and (3) participants have not participated in the same survey in other WeChat groups (participants might join different WeChat groups). Note that those who joined the emigration wave earlier and were not physically in China when they completed the survey would also be counted as valid responses, as long as they met the inclusion criteria above. The survey questions were displayed on the WeChat MiniApp *SurveyStar*. The researchers shared the study’s intent, informed consent, and survey link through the WeChat returnee public groups. The survey was in an anonymous manner and did not contain any information that could disclose the identities of the participants. All participants provided informed consent before participating in the survey.

After several rounds of screenings, the final valid sample included 1,014 responses. 455 out of 1,014 participants were male, accounting for approximately 44.87% of the total. There are 523 female responders, representing approximately 51.58%. Furthermore, 36 respondents, around 3.55% of the total, defined themselves as “Others” within this group. Among 1,014 participants, 61.74% were between 18 and 30 years old (626 people), 28.80% were in the 31–40 age range (292 participants), and 9.47% were in the 41–50 age range (96 participants). The studies involving human participants were reviewed and approved by the Institutional Review Board at China University of Political Science and Law, and relevant survey procedures were in line with the requirements of the Declaration of Helsinki. Written informed consent for participation in this study was provided by the participants. The data used in the study can be found at https://osf.io/vz425/.

The value of the variable *Acculturation* is the participant’s total score (ranging from 11–55) on the adapted Short Acculturation Scale (SAS) (Gupta and Yick, 2001) examining the acculturation of Chinese Americans. The original SAS was developed by Marin et al. (1987) and validated by several follow-up studies (Bai, 2016; Choi and Reed, 2011; Park et al., 2021). The questions in the SAS were adapted to the context of Chinese international student returnees in the present study, and SAS has also been used in a prior study on Chinese international students’ acculturation in a foreign environment (Cai, 2015). The variable *Worry* represents participants’ answers to the question “In general, how much do your concerns about migration or staying in China affect your mental health?” Answers are measured on a Likert scale ranging from “1” as none, all the way to “5” as “extremely.” Variables *ForDis* and *DomDis* are average scores on Perceived Discrimination Scale (PDS) measuring returnees’ perceived level of discrimination in the foreign country and China, respectively (Williams et al., 1997). The questions on the PDS were adapted to the context of Chinese international student returnees in the present study. The set of questions of the PDS was asked separately for both situations: foreign (when living abroad) and domestic (after returning to China). The variables *ForGenTrust* and *DomGenTrust* measure participants generalized trust towards the foreign society they lived in and Chinese society, respectively. Answers are measured on a Likert scale where “1” means none and “5” means extremely. Lastly, the variable *PerTrust* measures participants’ degree of personalized trust, with answers measured on a Likert scale where “1” means none and “5” means extremely (see Table 1).

**Table 1:** Variable description.

Variable name	Meaning	Value
Acculturation	The participant’s total score on the adapted Short Acculturation Scale	Ranging from 11 to 55
*Worry*	The participant’s degree of mental health impacts from concerns about staying in China or emigrating	1. None 2. A little 3. A considerable degree 4. Strongly 5. Extremely
*ForDis*	The participant’s average score on the Perceived Discrimination Scale while living in the foreign country	Ranging from 1 to 4
*DomDis*	The participant’s average score on the Perceived Discrimination Scale while living in China after returning from abroad	Ranging from 1 to 4
*ForGenTrust*	The participant’s degree of generalized trust toward the foreign society they were living in	1. None 2. A little 3. Moderately 4. Strongly 5. Extremely
*DomGenTrust*	The participant’s degree of generalized trust toward Chinese society	1. None 2. A little 3. Moderately 4. Strongly 5. Extremely
*PerTrust*	The participant’s degree of personalized trust	1. None 2. A little 3. Moderately 4. Strongly 5. Extremely

### 2.2 Analysis procedure

According to the aforementioned research objectives, two analytical models were constructed.

In Model 1, *Acculturation* is the outcome variable. Model 1 is as follows (see Equations 1–2).


(1) Acculturation~normal(μ,σ)

(2) $$\begin{array}{l} \mu_{i}=\beta_{0}+\beta_{\text{ForDis}}*{\text{ForDis}}_{i}+\beta_{\text{ForGenTrust}*\text{ForDis}}*\\ {\text{ForGenTrust}}_{i}*{\text{ForDis}}_{i}*+\\ \beta_{\text{PerTrust}*\text{ForDis}}*{\text{PerTrust}}_{i}*{\text{ForDis}}_{i} \end{array}$$

$\mu_{i}$ is the mean value of participant i ’s degree of foreign acculturation with posterior estimations in the form of normal distribution. Participant i ’s perceived foreign discrimination is measured by ${\text{ForDis}}_{i}$ . Participant i ’s generalized trust toward the foreign society is ${\text{ForGenTrust}}_{i}$ . Participant i ’s personalized trust is ${\text{PerTrust}}_{i}$ . Model 1 has an intercept $\beta_{0}$ and coefficients $\beta_{\text{ForDis}}$ , $\beta_{\text{ForGenTrust}*\text{ForDis}}$ and $\beta_{\text{PerTrus}*\text{ForDis}}$ . The logical network of Model 1 is visualized in Figure A1.

Model 2 was constructed as follows, with *Worry* being the outcome variable. Please see Equations 3, 4 as below.


(3) Worry~normal(μ,σ)

(4) $$\begin{array}{l} \mu_{i}=\beta_{0}+\beta_{\text{DomDis}}*{\text{DomDis}}_{i}+\beta_{\text{DomGenTrust}*\text{DomDis}}*\\ {\text{DomGenTrust}}_{i}*{\text{DomDis}}_{i}+\\ \beta_{\text{PerTrust}*\text{DomDis}}*{\text{PerTrust}}_{i}*{\text{DomDis}}_{i} \end{array}$$

Here, $\mu_{i}$ is the mean value of participant i ’s degree of mental health impacts from concerns about staying in China or emigrating with posterior estimations in the form of normal distribution. Participant i ’s perceived domestic discrimination is represented by ${\text{DomDis}}_{i}$ . Participant i ’s generalized trust toward domestic (Chinese) society is ${\text{DomGenTrust}}_{i}$ . Participant i ’s personalized trust is ${\text{PerTrust}}_{i}$ . Model 2 has an intercept $\beta_{0}$ and coefficients $\beta_{\text{DomDis}}$ , $\beta_{\text{DomGenTrust}*\text{DomDis}}$ and $\beta_{\text{PerTrust}*\text{DomDis}}$ . The logical network of Model 2 is visualized in Figure A4.

In the current research, we use Bayesian analysis with the aid of Markov Chain Monte Carlo (MCMC) algorithms to conduct the study. Bayesian analysis with the aid of MCMC has advantages in statistics especially when used on a relatively small sample size. Chinese international student returnees are a unique social group, so it is not likely to get a large sample size from this population. MCMC algorithms can generate a large number of simulated data points from the original data, increasing the accuracy of the estimation of the model’s posterior results. The Bayesian approach sees all characteristics probabilistically. Results are interpreted based on parameters with the highest probability of occurrence in their posterior distributions. This would increase the evaluation accuracy in psychological research (Csilléry et al., 2010; Dunson, 2001; Gill, 2014; Wagenmakers et al., 2018). The convergence of the Markov chain is checked via the indicators of effective sample size (*n_eff*) and the Gelman-Rubin shrink factor (*Rhat*). The *n_eff* values should be more than 1,000 (McElreath, 2020). The *Rhat* values should equal to 1 (Brooks and Gelman, 1998; Gelman and Rubin, 1992). The analysis was conducted via the bayesvl package in R (Vuong and La, 2019). Markov chain convergence visualization can also be processed by trace plots, Gelman-Rubin-Brooks plots, and autocorrelation plots. The MCMC configuration includes 5,000 total iterations, with Putnam, 2000 warm-up iterations and 4 chains.

## 3 Results

### 3.1 Effects of perceived foreign discrimination on acculturation: moderation by generalized and personalized trust

**Figure 1:**
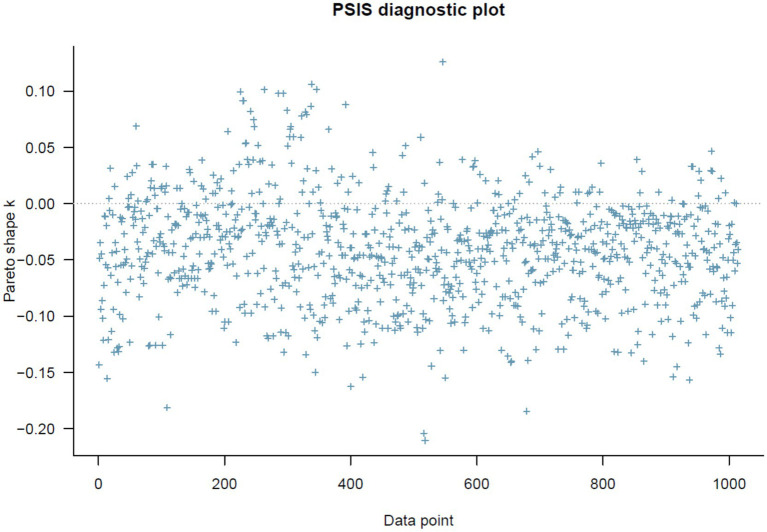
Model 1’s PSIS diagnostic plot.

This section evaluates RQ 1—how perceived discrimination abroad relates to acculturation and how generalized vs. personalized trust moderate that link. Here, the PSIS diagnostic plot below shows that all *k* values are lower than 0.5, indicating that Model 1 has a high goodness-of-fit with the current data (see Figure 1).

**Figure 2:**
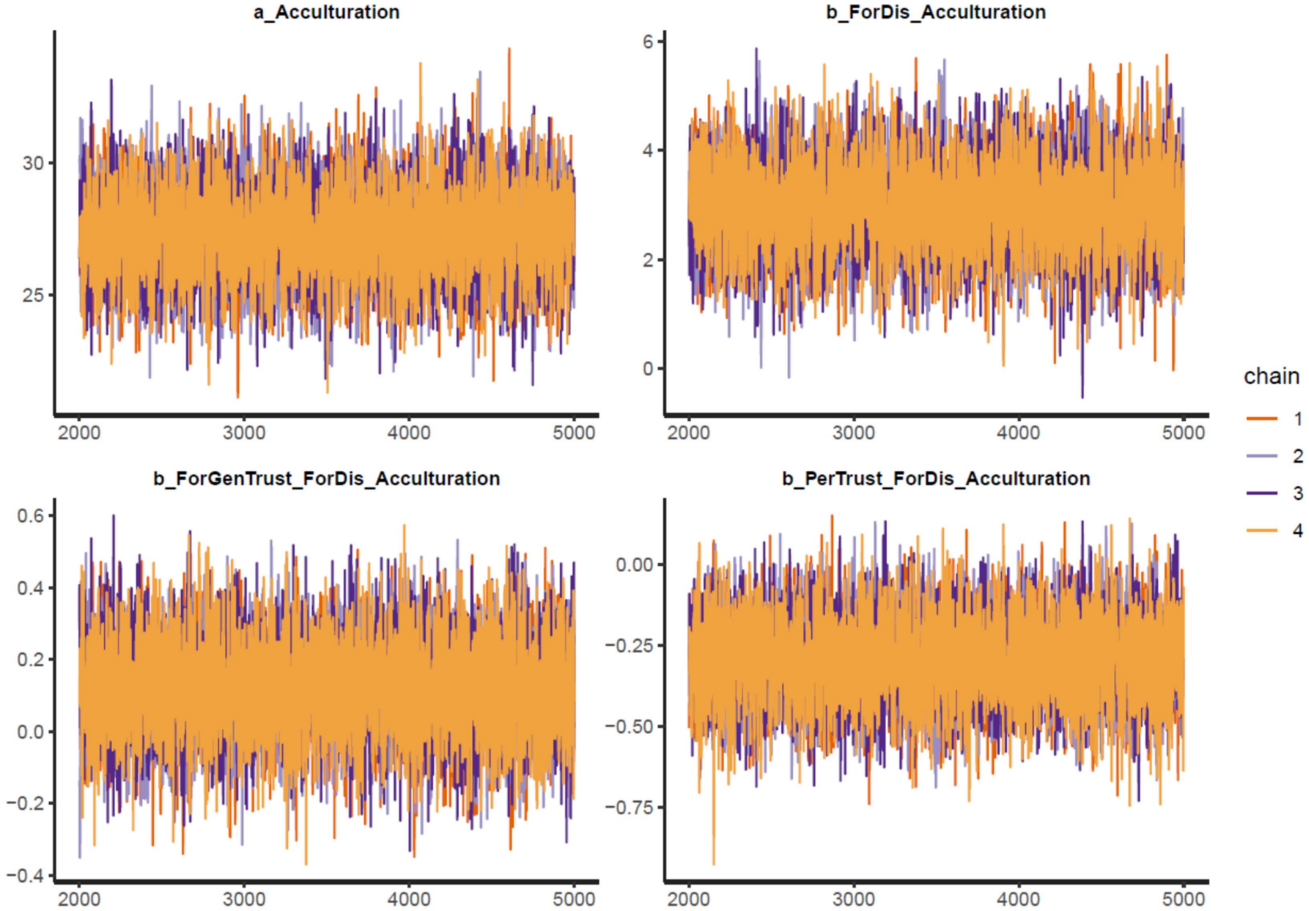
Model 1’s trace plots.

The results of Model 1 are illustrated in Table 2. All simulated posterior coefficients show good convergence of model 1’s Markov chains (see Table 2) based on the effective sample size (*n_eff* > 1,000) and Gelman-Rubin shrink factor (*Rhat* = 1). Figure 2 is Model 1’s trace plots, in which the colored lines represent the Markov chains. It can be observed that the fluctuation of lines occurs around a central equilibrium after 2,000 iterations (warmup period), suggesting it is well-mixed and of stationary qualities, which indicates it is a good convergence signal on the Markov chains. The Gelman-Rubin-Brooks plots show that *Rhat* values decrease shortly to 1 in the warm-up period (see Figure A2). The autocorrelation plots also indicate a fast elimination of problematic autocorrelation among simulated data points within the MCMC processes (see Figure A3).

**Table 2:** Model 1’s simulated posteriors.

Parameters	Mean (M)	Standard deviation (S)	n_eff	Rhat
Constant	27.33	1.66	7,472	1
*ForDis*	2.96	0.81	6,433	1
ForGenTrust*ForDis	0.12	0.14	9,527	1
PerTrust*ForDis	−0.29	0.13	7,928	1

**Figure 3:**
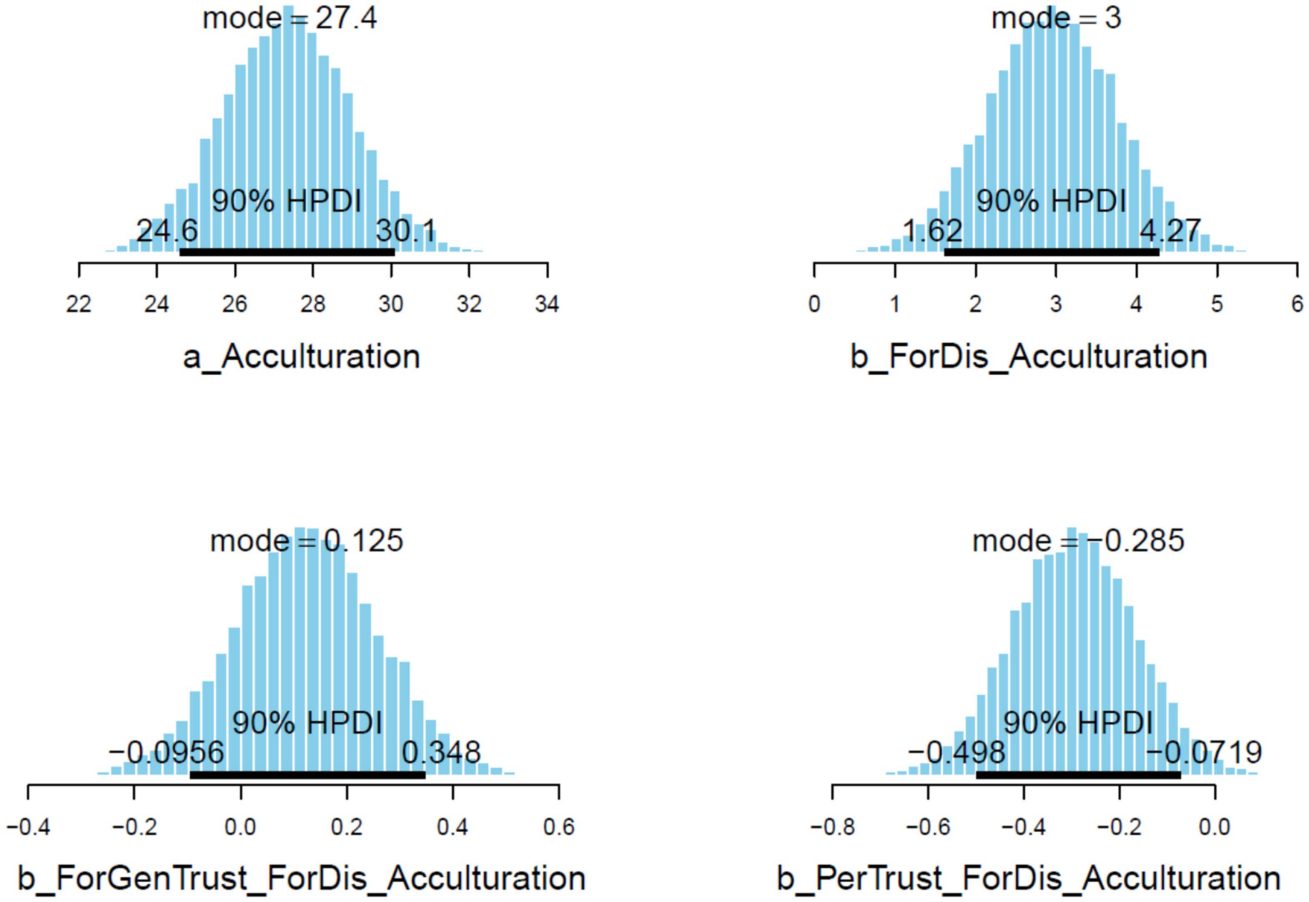
Model 1’s posterior distributions within 90% of highest posterior density intervals.

According to the results, *ForDis* has a clear positive association with Chinese international students’ level of acculturation ( $M_{\text{ForDis}}$ = 2.96 and $S_{\text{ForDis}}\;$ = 0.81). Meanwhile, generalized trust towards foreign society *ForGenTrust* has a positive moderating effect on this association (moderately reliable estimation) $(M_{\text{ForGenTrust}*\text{ForDis}}$ = 0.12 and $S_{\text{ForGenTrust}*\text{ForDis}}$ = 0.14). Personalized trust *PerTrust* has a clear negative moderating effect on the relationship $(M_{\text{PerTrust}*\text{ForDis}}$ = − 0.29 and $S_{\text{PerTrust}*\text{ForDis}}\;$ = 0.13). In Figure 3, it can be observed that the posterior distributions of *ForDis* are entirely on the positive side, and ForGenTrust*ForDis are mostly on the positive side, whereas PerTrust*ForDis are mostly on the negative side.

### 3.2 Effects of perceived domestic discrimination on reintegration worry: moderation by generalized and personalized trust

**Figure 4:**
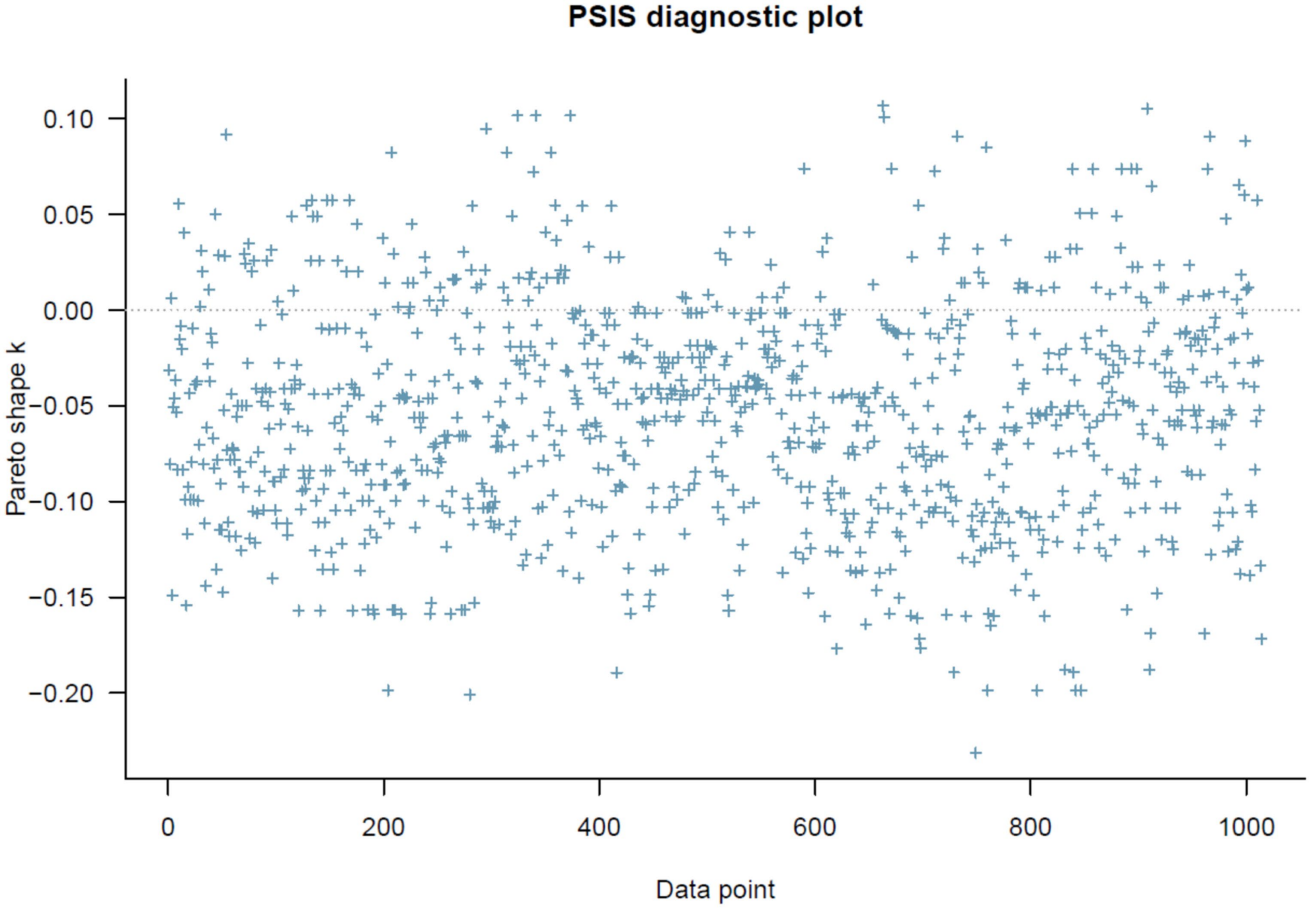
Model 2’s PSIS diagnostic plot.

Here we test RQ 2—whether discrimination back in China predicts mental-health-related worries about staying vs. emigrating, and the moderating roles of trust. The PSIS diagnostic plot below indicates that all *k* values are lower than 0.5, indicating that Model 2 has a high goodness-of-fit with the current data (see Figure 4).

**Figure 5:**
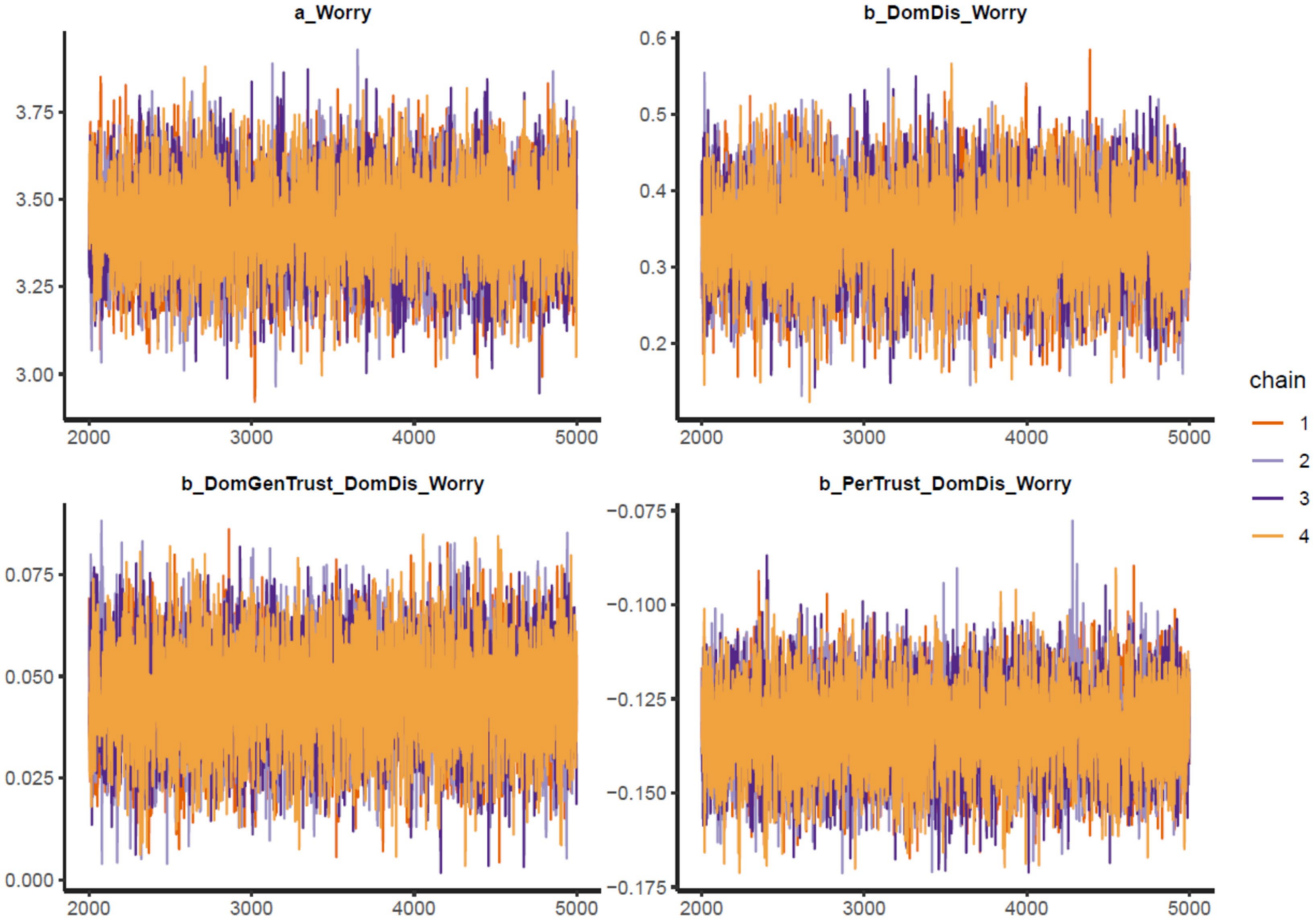
Model 2’s trace plots.

The results of Model 2 are illustrated in Table 3. All simulated posterior coefficients show good convergence of model 2’s Markov chains (see Table 3) based on the effective sample size (*n_eff* > 1,000) and Gelman-Rubin shrink factor (*Rhat* = 1). Figure 5 is Model 2’s trace plots. Similar to the explanation in Model 1, the trace plots here also indicate good convergence of the Markov chains. The Gelman-Rubin-Brooks plots and the autocorrelation plots for Model 2 also show signs of good statistical reliability (see Figures A5, A6).

**Table 3:** Model 1’s simulated posteriors.

Parameters	Mean (M)	Standard deviation (S)	n_eff	Rhat
Constant	3.43	0.13	8,235	1
*DomDis*	0.34	0.06	7,480	1
DomGenTrust* DomDis	0.05	0.01	9,324	1
PerTrust*DomDis	−0.13	0.01	9,869	1

**Figure 6:**
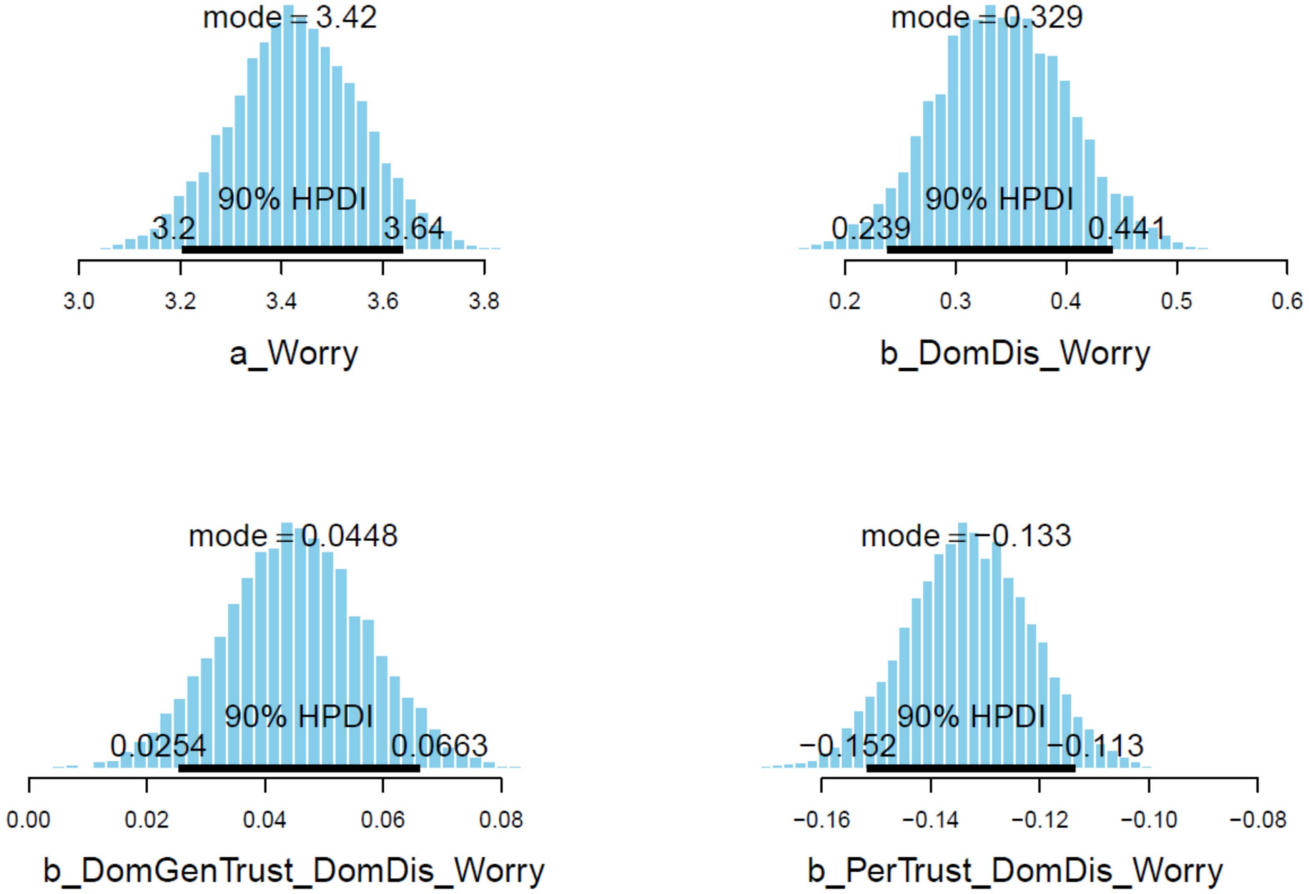
Model 2’s posterior distributions within 90% of highest posterior density intervals.

According to the results, perceived domestic discrimination from Chinese society *DomDis* has a clear positive association with Chinese returnees’ negative mental health impacts due to concerns about staying in China or emigrating ( $M_{\text{DomDis}}$ = 0.34 and $S_{\text{DomDis}}\;$ = 0.06). Generalized trust towards domestic society *DomGenTrust* has a clear positive moderating effect on this association $(M_{\text{DomGenTrust}*\text{DomDis}}$ = 0.05 and $S_{\text{DomGenTrust}*\text{DomDis}}$ = 0.01). Personalized trust *PerTrust* has a clear negative moderating effect on the relationship $(M_{\text{PerTrust}*\text{DomDis}}$ = − 0.13 and $S_{\text{PerTrust}*\text{DomDis}}\;$ = 0.01). In Figure 6, it can be observed that the posterior distributions of *DomDis* and DomGenTrus*DomDis are entirely on the positive side, and PerTrust*DomDis are entirely on the negative side.

## 4 Discussion

The analysis results show that, in a foreign country’s environment, interestingly, the degree of acculturation and perceived discrimination has a positive association. Generalized trust positively moderates this association (moderately reliable estimation), while personalized trust has a negative moderating effect. In the domestic environment of China, perceived discrimination is positively associated with the degree of mental health problems induced by concerns about whether to stay in China or migrate away. Generalized trust positively moderates this association, while personalized trust negatively moderates it. In both environments, the magnitude of the moderating effect of personalized trust is higher than that of generalized trust.

### 4.1 The multi-layered influences of perceived discrimination and trust on social integration

The positive correlation between perceived discrimination and higher acculturation degree in the foreign social environment is an interesting result, which may seem a little strange at first glance and inconsistent with prior research findings regarding the negative association between discrimination and social integration (Okoye et al., 2023; Topa et al., 2023). However, when considering the realistic experiences of international students living in a foreign country, we can have explanations from both possible directions of the relationship. Among various aspects of acculturation, language and cultural proficiency, and social connections are two major factors (Hammer, 2017; Oppedal et al., 2004; Wang, 2023; Yoon et al., 2012). As international students become more proficient in the foreign language, they can also pick up more verbal and cultural practice nuances related to discrimination that low-language/culture-proficiency ones may not be aware of Gong et al. (2017). The expansion of social connections in the process of acculturation also increases the probability of being exposed to discriminative treatment, including in direct interactions with new encounters or through stories relayed by new acquaintances (Yoon et al., 2012). After all, when international students step out of college campuses to explore the host societies, it has been reported that international students are more likely to encounter racism and discrimination off campus than on campus (Hanassab, 2006). In the other possible direction of influence, perceived discrimination has been proven to induce a stronger desire to try to conform to the standard of the foreign collective (Branscombe et al., 1999; Yip et al., 2008). For many Chinese international students, many seek “cosmopolitan imagery”—referring to international students’ envisioning of an expansion of their perspectives, development transnational identities, and eliminations of both ethnocentrism and micro-aggressions directed at different national and cultural groups (Nam et al., 2024). Previous studies have proven that a strong sense of self-affirmation and autonomy (in this case, cosmopolitan imagery-seeking mentality) can reduce individual defensiveness, opening individuals to engaging more constructively with social adversity, even shifting perceptions of racism depending on context and group identity (Adams et al., 2006; Sherman and Cohen, 2002). Therefore, those who are in pursuit of cosmopolitan imagery could see this as a challenge, promoting greater efforts to fit in. This is also in alignment with the evolutionary standpoint on social conformity rooted in natural survival pressure for humans as a social species (Berns et al., 2005; Boyd and Richerson, 1982; Claidière and Whiten, 2012; Konrad and Morath, 2012).

In the domestic social environment after returning, the positive correlation between perceived discrimination and the degree of mental health problems induced by concerns about whether to stay in China or migrate away is a rather intuitive result. Here, due to the nature of perceived mental burden being on the outcome side of related psychological processes, we can interpret an influence direction from perceived discrimination to the likelihood of such worries. Being discriminated against upon returning to one’s home country can feel like a betrayal in the minds of returnees, causing mental distress among returnees (Jin et al., 2024b). Unfortunately, the political and socioeconomic situations of China in the post-COVID-19 time may further escalate the negative perceptions from the collective towards returnees, and vice versa (Bhattacharya, 2019; Catalano and Wang, 2021; Jin and Wang, 2022; Leutert, 2018; Tao, 2021). Unlike the case of foreign society, Chinese returnees already possess their native language proficiency and pre-established social networks in China. Thus, conflicts of values within one’s mind can lead to confusion about the sense of belonging and corresponding behavioral planning or action (Jin and Wang, 2022). These concerns in terms of social reintegration are expressed as a higher degree of burden on one’s mental health about settling on a suitable living environment in the near future – whether to stay or to leave.

Generalized trust and personalized trust influence the relationship between perceived discrimination and social integration differently. In the case of foreign society, generalized trust may help increase the volume of the information channels between the individual and other interacting people (Putnam, 2000; Quigley, 1996). This in turn can lessen the negative meanings attached to observations of perceived discrimination and is consistent with prior research on the link between generalized trust and discrimination (Nannestad, 2008; Uslaner, 2002; Welch et al., 2005). Generalized trust in itself is also inherently a form of positive meaning toward the general public, which directly helps increase the willingness to integrate into said collective (Portes and Sensenbrenner, 1993). Personalized trust, on the other hand, indicates a higher capacity of information channels connected to one’s close social circle due to the higher default perceived value of received information from such contacts. In the context of Chinese international students, a strongly attached close social circle may exhibit the ingroup-outgroup effect (Tajfel, 1978; Tajfel and Turner, 1979). Due to the highly reinforced ingroup’s collective perceptions, perceived discrimination from the outgroup (here: the foreign society) is heightened and its negative implications are exacerbated. This line of thought is also referred to as an “echo chamber” in communication studies (Ulen, 2001), aligning with former studies on the effect of group mentality on discrimination, especially racial discrimination between the host country and immigrant groups (Brewer, 1999; Delhey and Newton, 2005; Nyhan and Reifler, 2010; Simonsen, 2016; Uslaner, 2017; Yamagishi and Yamagishi, 1994).

In Chinese society, especially urban areas, social trust is generally perceived to be low due to various reasons (Dong et al., 2018; Jiang et al., 2020). For Chinese international students with transnational in-between identities, those leaning towards the China side might inherit such a social reality to exhibit lower social trust toward strangers and the public in Chinese society. Conversely, students who demonstrate a stronger trust in the domestic public (especially towards strangers) may indicate a greater level of acculturation in the West during their overseas studies. After all, Chinese public has a lower social trust level compared to the West (Torpe and Lolle, 2011). Now that Chinese society has shifted towards cultural conservatism, reflected by a more aggressive diplomatic stance, centralized government control, and labor rights infringement amid low economic growth (Blanchette and Medeiros, 2022; CEIC, 2024; Iida, 2020; IMF, 2023; Zhao, 2023), upon returning to China, the highly acculturated and liberalized returnees are facing such a social reality that is in sharp contrast to their transnational identity formed overseas. Prior studies have indicated that mental distresses such as identity conflicts, cognitive dissonances, and perceived stigmatization by the public can cause severe mental health issues among Chinese returnees (Jin et al., 2024a; Jin et al., 2024b; Jin et al., 2024c; Jin et al., 2024d), especially during the escalation of cybernationalism and xenophobic sentiments. When these sentiments eventually turned into two hate crimes against foreigners in 2024 (AP News, 2024; CNN, 2024), liberalized returnees’ mental health conditions could be worsened. In addition, they might feel a higher level of betrayal, and thus increase their worries about whether they should reintegrate or choose the migration option (Jin et al., 2024c; Jin et al., 2024b), because in this case, the general public is their own country’s people to which they have some form of national and racial identity attachment. And this carries different mental values compared to the people of a foreign country (Klest et al., 2019; Platt and Freyd, 2015). Personalized trust, on the other hand, is suggested to mitigate the negative effect of domestic discrimination on returnees’ reintegration concerns. A strong positive connection to one’s close social circle within one’s own country may help shelter from or buffer the negativity of discrimination from the public (Dailey et al., 2023; Kawachi and Berkman, 2001). Unlike the case of migrant ingroup living in a foreign country which likely reinforces discriminative perceptions, a close social circle of one’s home country belongs to the same national collective group, offering social support from peers, friends, and family, which has been proven effective in mitigating culture shock/reverse culture shock’s impact (such as value conflicts in returnees’ minds) on individual’s mental distress (Furnham, 2019; Li and Peng, 2019; Sippel et al., 2015).

**Figure 7:**
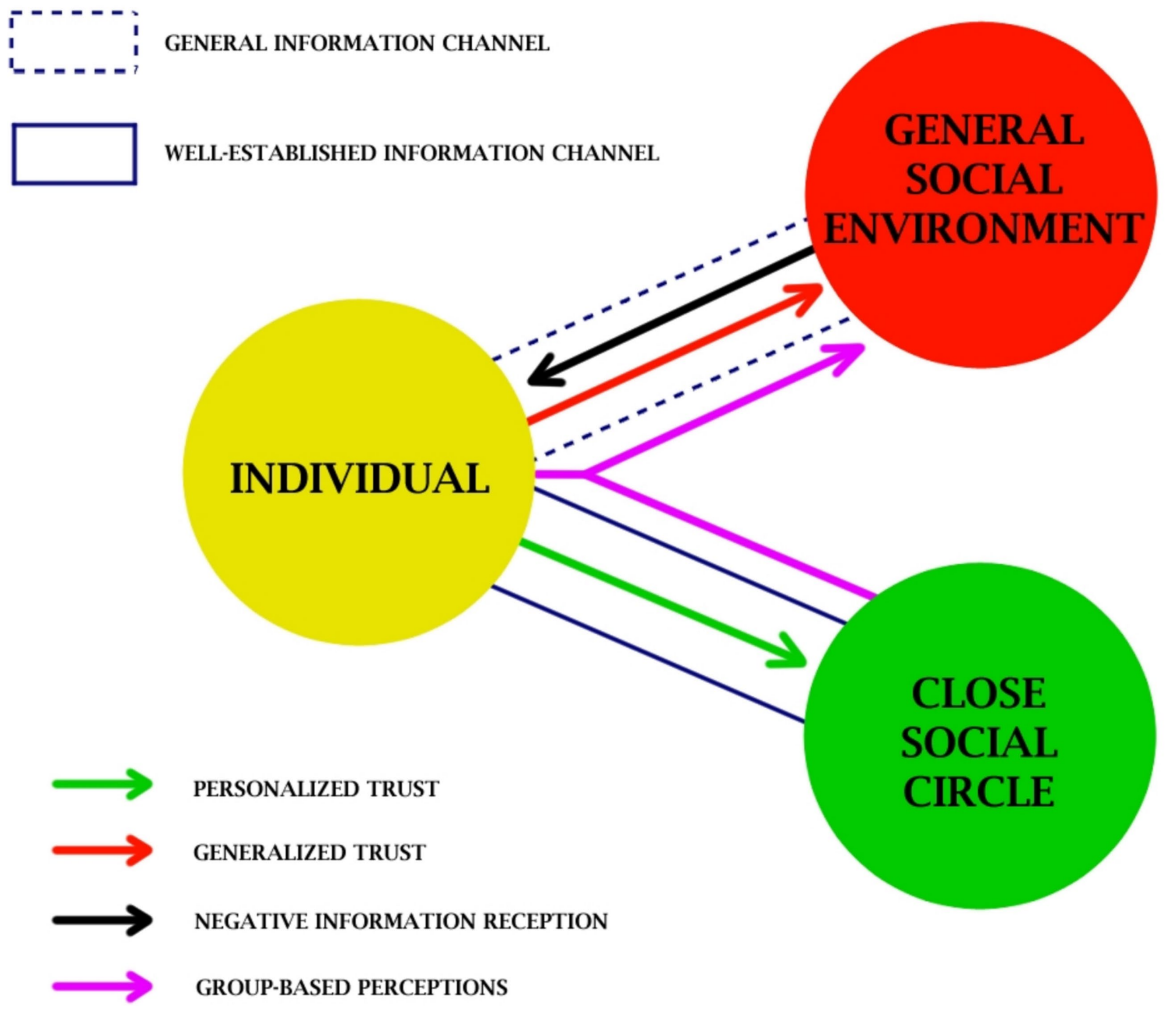
Visualization of the information interactions related to two different types of trust.

Figure 7 helps visualize the information interactions in the scenarios of this study. Here, trust serves as a form of information reception compatibility – in other words, higher receptivity toward information coming from the corresponding trusted source. Thus, trust can serve as a psychological mechanism for expanding or strengthening the information channels between the individual and the source. Note that for the case of generalized trust (red arrow), such channels are often temporary and encounter-based (dotted blue frame), carrying a low or negligible degree of social commitment. Meanwhile, personalized trust (green arrow) is applied to well-established channels (lined blue frame), often accompanied by relationship commitment, emotional attachment, and individual responsibility. In the case of foreign society, the close social circle and the general social environment would have little overlap; while in the case of domestic society, the close social circle would lie almost completely within the general social environment. Our study shows how the non-linear information flow can cause group-based perceptions to affect how individuals react differently to the negative received information from the general public (in this case: perceived discrimination).

### 4.2 Implications

The findings from this study have several important implications for understanding the experiences of Chinese international student returnees in both foreign and domestic contexts, especially in the wake of the COVID-19 pandemic. First of all, given the nuanced role of personalized trust, it is suggested that educational institutions overseas might consider offering diverse and integrative orientation programs rather than simply relying on co-national-based social networks such as Chinese student alumni or associations to help Chinese international students smooth their integration, which has been substantiated by prior studies (Bender et al., 2019; Cao et al., 2018). On the other hand, Chinese colleges and workplaces should offer more mentor-mentee programs, along with other reentry support programs to bridge the cultural gap between returnees and domestic workers to lower the mental distress during their reintegration. Prior research has indicated the need and effectiveness of such programs for youth during orientation and acculturation (Arouca, 2013; DuBois et al., 2011). Lastly, the findings suggest that generalized trust plays a significant role in how individuals navigate social integration and respond to discrimination. Policy makers can therefore prioritize fostering environments that promote generalized trust—such as through transparent governance, inclusive public discourse, and equitable institutional practices—to enhance social cohesion and reduce the adverse impact of discrimination on returnees. Building generalized trust not only supports individual psychological well-being and adaptive coping but also contributes to a more resilient, inclusive, and globally engaged society in the post-pandemic era. Further research into strategies to build and maintain generalized trust within communities could offer additional insights into improving the experiences of international students and returnees.

### 4.3 Limitations

The study is not without limitations. First of all, the study is based on an online survey of WeChat users, which inevitably excluded those returnees who do not use WeChat as their social media platform. The present study examined self-reported data, which might be subject to some degree of personal biases, especially regarding sensitive topics such as discrimination, trust, and emigration, given the sociopolitical contexts that the participants might have consciously or subconsciously considered. Furthermore, regarding the multifaceted psychological pathways of interpersonal relationships, qualitative studies, longitudinal approaches, and psychosocial experiments are needed to better understand the complex interactions between perceived discrimination, trust, and acculturation. Samples of populations from other regions/countries will also help update the patterns found in the present study. Furthermore, our data were collected between October 2023 and January 2024—by which time most COVID-19 restrictions had been lifted—so the discrimination and trust dynamics we observed may underrepresent the acute, crisis-driven reentry challenges (e.g., strict quarantines, heightened xenophobia) faced by those who returned during the 2020–22 peak. We also did not stratify respondents by their year of return (e.g., early-2020 vs. late-2023 cohorts), limiting our ability to examine how reacculturation experiences evolved across pandemic stages. Future research should use stratified or longitudinal designs, as well as qualitative interviews to offer deeper insights into this research topic.

## Data Availability

The original contributions presented in the study are publicly available. This data can be found here: https://osf.io/vz425/.
