# Supplementary material for: How perceived discrimination and trust dynamics influence social integration in acculturation and reacculturation: the case of Chinese international student returnees
Source: Front Psychol. 2025 May 15;16:1597967. doi: 10.3389/fpsyg.2025.1597967 (PMC12123363; doi:10.3389/fpsyg.2025.1597967)
Supplement: Supplementary file 1 [file Supplementary_file_1.docx]

**Survey design**

**1. Demographic information**

1.1. What is your self-identified gender?

| Male | Female | Other |
| --- | --- | --- |

1.2. What is your age group?

| <18 | 18-30 | 31-40 | 41-50 | >50 |
| --- | --- | --- | --- | --- |

1.3. What is your highest educational attainment?

| Elementary or lower | Secondary School | High School | Undergraduate | Postgraduate or higher |
| --- | --- | --- | --- | --- |

1.4. How long have you spent your life in a foreign country/countries?

| <1 year | 1-2 years | 2-5 years | 5-10 years | >10 years |
| --- | --- | --- | --- | --- |

**2. Intention and identity cluster**

2.1. Have you ever considered emigrating and, if so, how strong is your desire to do so?

| Definitely stay | Likely stay | Unsure | Likely migrate | Definitely migrate |
| --- | --- | --- | --- | --- |

2.2. What is the main reason why you intend to stay in China or migrate?

| Education | Career/Profession | Living with family or loved ones | Financial constraint/motivation | Cultural values |
| --- | --- | --- | --- | --- |

2.3. How do you identify yourself as one of the following?

Homestayers prefer spending time with other Chinese students and engaging in familiar activities from home. They feel a strong connection to my home culture and less identification with the foreign culture of my study abroad location. They are hesitant to explore or engage much with the local culture.

Navigator have mixed emotional attachments to both my home culture and the foreign culture of my study abroad location. They flexibly interact with norms and values from both cultures. They have a high degree of flexibility and ability to adjust my behaviors between cultures. They pursue transnational strategies to build connections in both places.

Wayfarers feel disconnected from home culture and are critical/skeptical of it. They are motivated to explore the foreign culture of my study abroad location. They feel a strong connection and sense of belonging to the new foreign culture. They are eager to have new experiences and adventures abroad. They don't have a clear plan but want to embed myself transnationally.

| Homestayer | Navigator | Wayfarer |
| --- | --- | --- |

**3. Acculturation**

Short Acculturation Scale (SAS)

Questions (have been adapted for this returnee survey):

Think about when you were or will be in the foreign country:

3.1. In general, what language(s) do you read and speak?

3.2. What was the language(s) you used as a child?

3.3. What language(s) do you usually speak at home?

3.4. In which language(s) do you usually think?

3.5. What language(s) do you usually speak with your friends?

3.6. In what language(s) do you usually consume (watch or listen to) media content?

3.7. In general, in what language(s) do you *prefer* to consume media content?

| Only Chinese | More Chinese than non-Chinese | Both equally | More non-Chinese than Chinese | Only non-Chinese |
| --- | --- | --- | --- | --- |

3.8. Your close friends are:

3.9. You prefer to go to social gatherings/parties at which the people are:

3.10. The persons you visit or who visit you are:

3.11. If you could choose your children’s friends, you would want them to be:

| All Chinese | More Chinese than non-Chinese | About half and half | More non-Chinese than Chinese | All non-Chinese |
| --- | --- | --- | --- | --- |

**4. Current mental health status**

4a. [Standard Chinese PHQ-9]

Over the last two weeks, how often have you been bothered by any of the following problems?

| Not at all (0) | Several days (1) | More than half the days (2) | Nearly every day (3) |
| --- | --- | --- | --- |

4.1. Little interest or pleasure in doing things

4.2. Feeling down, depressed, or hopeless

4.3. Trouble falling or staying asleep, or sleeping too much

4.4. Feeling tired of having little energy

4.5. Poor appetite or overeating

4.6. Feeling bad about yourself – or that you are a failure or having let yourself or your family down

4.7. Trouble concentrating on things, such as reading the newspaper or watching television

4.8. Moving or speaking so slowly that other people could have noticed. Or the opposite – being so fidgety or restless that you have been moving a lot more than usual

4.9. Thoughts that you would be better off dead, or of hurting yourself in some way

Total score:

4.10. In general, how much do your concerns about migration or staying in China affect your mental health?

| None | A little | A considerable degree | Strongly | Extremely |
| --- | --- | --- | --- | --- |

**5. Discrimination**

[Q12-20 from the Perceived Discrimination Scale]

| Never (0) | Rarely (1) | Sometimes (2) | Often (3) |
| --- | --- | --- | --- |

In general, how did you feel when you were living in the foreign country/countries?

5.1.1. You are treated with less courtesy than other people.

5.1.2. You are treated with less respect than other people.

5.1.3. You receive poorer service than other people at restaurants or stores.

5.1.4. People act as if they think you are not smart.

5.1.5. People act as if they are afraid of you.

5.1.6. People act as if they think you are dishonest.

5.1.7. People act as if they think you are not as good as they are.

5.1.8. You are called names or insulted.

5.1.9. You are threatened or harassed.

In general, how did you feel being in China after returning from abroad?

5.2.1. You are treated with less courtesy than other people.

5.2.2. You are treated with less respect than other people.

5.2.3. You receive poorer service than other people at restaurants or stores.

5.2.4. People act as if they think you are not smart.

5.2.5. People act as if they are afraid of you.

5.2.6. People act as if they think you are dishonest.

5.2.7. People act as if they think you are not as good as they are.

5.2.8. You are called names or insulted.

5.2.9. You are threatened or harassed.

**6. Other psychosocial aspects**

| None | A little | Moderately | Strongly | Extremely |
| --- | --- | --- | --- | --- |

6.1. In general, how much do you trust people in Chinese society, including strangers?

6.2. In general, how much do you trust people in the host country you stayed, including strangers?

6.3. In general, how much do you trust people with established personal connections (e.g. family members, close friends, loved ones, etc.)?

6.4. How much do you embrace individualism (as opposed to collectivism)?

6.5. In general, how much are you interested in politics?

6.6. How much do you consider yourself as a spiritual person?

6.7. How much do you believe in fate/destiny?

6.8. How much do you think that changes in living conditions/environments can bring happiness or fulfillment?
